# Population pharmacokinetic analysis of teicoplanin in paediatric patients, including those receiving continuous kidney replacement therapy: a prospective cohort study

**DOI:** 10.1093/jac/dkaf012

**Published:** 2025-01-23

**Authors:** Laura Butragueño-Laiseca, Gastón García-Orueta, Natalia Riva, Iñaki F Trocóniz, Sarah N Fernández, Verónica Camacho Vicente, Belén Padilla, María Slöcker, María José Santiago

**Affiliations:** Pediatric Intensive Care Unit, Hospital General Universitario Gregorio Marañón, Madrid, Spain; Instituto de Investigación Sanitaria Gregorio Marañón (IiSGM), Madrid, Spain; Departamento de Salud Departamento de Salud Pública y Materno Infantil, Universidad Complutense de Madrid, Madrid, Spain; Red de Investigación Cooperativa Orientada a Resultados en Salud (RICORS RD21/0012/0011), Institute of Health Carlos III, Madrid, Spain; Department of Pharmaceutical Sciences, School of Pharmacy and Nutrition, University of Navarra, Pamplona, Spain; Department of Pharmaceutical Sciences, School of Pharmacy and Nutrition, University of Navarra, Pamplona, Spain; Navarra Institute for Health Research (IdiSNA), Pamplona, Spain; Department of Pharmaceutical Sciences, School of Pharmacy and Nutrition, University of Navarra, Pamplona, Spain; Navarra Institute for Health Research (IdiSNA), Pamplona, Spain; Institute of Data Science and Artificial Intelligence, DATAI, University of Navarra, Pamplona, Spain; Pediatric Intensive Care Unit, Hospital General Universitario Gregorio Marañón, Madrid, Spain; Instituto de Investigación Sanitaria Gregorio Marañón (IiSGM), Madrid, Spain; Departamento de Salud Departamento de Salud Pública y Materno Infantil, Universidad Complutense de Madrid, Madrid, Spain; Red de Investigación Cooperativa Orientada a Resultados en Salud (RICORS RD21/0012/0011), Institute of Health Carlos III, Madrid, Spain; Pediatric Intensive Care Unit, Hospital General Universitario Gregorio Marañón, Madrid, Spain; Instituto de Investigación Sanitaria Gregorio Marañón (IiSGM), Madrid, Spain; Departamento de Salud Departamento de Salud Pública y Materno Infantil, Universidad Complutense de Madrid, Madrid, Spain; Red de Investigación Cooperativa Orientada a Resultados en Salud (RICORS RD21/0012/0011), Institute of Health Carlos III, Madrid, Spain; Instituto de Investigación Sanitaria Gregorio Marañón (IiSGM), Madrid, Spain; Clinical Microbiology Department, Hospital General Universitario Gregorio Marañón, Madrid, Spain; Pediatric Intensive Care Unit, Hospital General Universitario Gregorio Marañón, Madrid, Spain; Instituto de Investigación Sanitaria Gregorio Marañón (IiSGM), Madrid, Spain; Departamento de Salud Departamento de Salud Pública y Materno Infantil, Universidad Complutense de Madrid, Madrid, Spain; Red de Investigación Cooperativa Orientada a Resultados en Salud (RICORS RD21/0012/0011), Institute of Health Carlos III, Madrid, Spain; Pediatric Intensive Care Unit, Hospital General Universitario Gregorio Marañón, Madrid, Spain; Instituto de Investigación Sanitaria Gregorio Marañón (IiSGM), Madrid, Spain; Departamento de Salud Departamento de Salud Pública y Materno Infantil, Universidad Complutense de Madrid, Madrid, Spain; Red de Investigación Cooperativa Orientada a Resultados en Salud (RICORS RD21/0012/0011), Institute of Health Carlos III, Madrid, Spain

## Abstract

**Objectives:**

Teicoplanin is a commonly used antibiotic in critically ill children. However, teicoplanin dosing is often inaccurate, especially in children undergoing continuous kidney replacement therapy (CKRT). This study aims to develop a population pharmacokinetic (PK) model to optimize teicoplanin dosing in critically ill children, including those on CKRT.

**Methods:**

Data from 26 critically ill children (12 with CKRT) receiving the standard dosing regimen were analysed. In total, 172 teicoplanin concentration measurements from plasma, pre- and post-filter ports were modelled simultaneously using NONMEM 7.4. Simulations were conducted to assess the target attainment (*C*_min _= 10 mg/L and AUC_24_/MIC > 800 h) of the current standard dosing regimen and of different alternative dosing regimens.

**Results:**

A two-compartment model was selected. Weight significantly affected renal clearance and volume of distribution of the central compartment, while filter surface area affected haemofilter clearance. Only 16 patients (59%) achieved a *C*_min_ of >10 mg/L with the standard dosing regimen, and only 1 achieved the target AUC/MIC. Based on simulation results, 3 × 15 mg/kg q12h + 10 mg/kg q24h (CKRT) and 3 × 15 mg/kg q12h + 15 mg/kg q24h (no CKRT) could be better alternative regimens.

**Conclusions:**

This population model is a good proof of concept to develop modelling approaches that could help in an individualized dosing approach that needs to be adopted in critically ill paediatric patients. The standard paediatric dosage for teicoplanin could be insufficient for optimal exposure, and higher doses may benefit both CKRT and non-CKRT patients.

## Introduction

Patients in ICUs have a high risk of infection by Gram-positive pathogens, especially by MRSA.^1,2^ The efficacy of teicoplanin has been demonstrated against a range of infections. These include MRSA, CoNS, *Enterococcus* spp., *Streptococcus* groups A, B, C and G, *Streptococcus pneumoniae* and viridans-group streptococci.^3,4,5^

Existing evidence indicates that plasma concentrations of antibiotics are outside the therapeutic range in a substantial proportion of adult (41%) and paediatric (95%) critically ill patients.^6^ Moreover, a significant proportion of children in ICUs, around one in four, develop acute kidney injury (AKI), with over 10% requiring continuous kidney replacement therapy (CKRT).^7^ To the best of our knowledge, there are no studies characterizing the pharmacokinetics of teicoplanin in paediatric patients undergoing CKRT. Therefore, dosing individualization in these patients often relies on extrapolations from either adult CKRT patients or non-critically ill children.^2,8^

Teicoplanin, a glycopeptide antibiotic primarily utilized against MRSA infections, exhibits therapeutic efficacy closely linked to its plasma trough concentration (*C*_min_). The therapeutic target often consists of a *C*_min_ value of 10 mg/L,^9^ although higher trough concentrations of 15 or 20 mg/L can be desired in severe infections. However, some recent studies propose that efficacy can also be determined by the AUC/MIC ratio, where a value of >800 is considered optimal.^10,11^ Regarding its PK characteristics, teicoplanin is a hydrophilic compound with an apparent volume of distribution higher than the blood volume, highly (>90%) bound to albumin, and eliminated mainly by renal excretion.^12^

The lack of studies in teicoplanin involving paediatric patients undergoing CKRT might lead to inaccurate dosing, resulting in insufficient drug exposure, antibiotic resistance and delayed recovery. Therefore, the aim of this study was the development of a PK model describing teicoplanin exposure in this specific patient population and to explore if any patient characteristics are related to improving precision dosing of teicoplanin.

## Methods

This study was part of a broader project focused on characterizing the PK properties of different antibiotics administered to paediatric patients undergoing CKRT (European Regional Development Fund, ref. RD16/0022/0007). The eligibility criteria for the study were patients admitted to paediatric ICU (PICU), up to the age of 16 years, with a venous cannula that allowed needle-free blood sampling.

### Ethics

The study received approval in May 2017 (record number 10/2017) from the Gregorio Marañón Institutional Review Board, in Madrid, Spain. Informed consent was obtained from the parents of all participating patients. Written informed consent was obtained from parents and children over 12 years old.

### Dosing, sampling and analytical method

Patients received the current standard dosing regimen, consisting of three loading doses of 10 mg/kg/12 h in all cases, followed 24 h later by 10 mg/kg/24 h in patients without haemofilter or 3.3 mg/kg/24 h in patients undergoing CKRT.^13^ Teicoplanin was administered via IV infusion over 5 min. Blood samples were collected using an IV catheter, enabling needle-free blood extraction in the PICU. In general, sampling began after patients had received at least three doses, although in four patients sampling began after receiving the first two doses. Blood samples (1 mL) were collected in heparinized tubes before and 1, 6, 12 and 24 h after the start of the infusion. In patients with CKRT, blood samples were drawn simultaneously from the pre- and post-filter ports of the Prismaflex^©^ (Baxter Int.) device. The median (range) number of samples obtained for each patient was 5.5 (4–13). Blood samples were first centrifuged at 2500 rpm for 10 min to produce a minimum of 350 µL of plasma, and were then preserved at −80°C. Teicoplanin concentrations were measured with a validated liquid chromatography with tandem mass spectrometry. The analytical method is detailed in the Supplementary material (available as Supplementary data at *JAC* Online).

### PK analysis

The population approach was employed to simultaneously analyse all the available data from all patients (with and without haemofilter), including plasma, pre-filter and postfilter concentrations. The analyses were conducted using NONMEM 7.4,^14^ utilizing the first-order conditional estimation method.

For the analysis, teicoplanin concentrations were logarithmically transformed. Inter-individual variability (IIV) was described with the exponential model and residual variability was described with the additive error model in the logarithmic scale, and different magnitudes of residual error were estimated for each type of measured concentrations [plasma/pre-filter (*C*_Pre_) and post-filter (*C*_Post_)]. A detailed description of the model building, selection and evaluation is provided in the Supplementary material.

#### Percentage of target attainment

Table S1 lists the different dosing regimens evaluated. For each of those regimens, the percentage of simulated patients showing a value of: (i) *C*_min_ at 72 h post initiation of therapy (after the first maintenance dose) of 10, 15 or 20 mg/L; or (ii) AUC_24_/MIC > 800 derived for MIC values of 0.25, 0.5, 1, 2, 4 and 8 mg/L, where AUC_24_ represents the area under the plasma concentration-versus-time curve obtained between 48 and 72 h post initiation of teicoplanin therapy (after the first maintenance dose), were calculated and considered as the percentage of target attainment.

For each clinical scenario (dosing regimen and type of patient), 1000 simulated plasma concentration-versus-time profiles were simulated for the following body weight groups: 3–10, 11–30 and 31–60 kg (see Figure S1). A threshold trough concentration for treatment toxicity (*C*_min _= 60 mg/L)^15^ was considered as an additional criterion to choose the most adequate therapeutic regimen.

## Results

### Patient population

Twenty-six critically ill children treated with teicoplanin were enrolled in the study, 12 of them undergoing CKRT in continuous venovenous haemodiafiltration modality. They showed a median (range) weight and age of 8 kg (4.3–44 kg) and 14 months (3 months–13 years), respectively. There were no statistically significant differences between the two groups in age, weight or height (*P* > 0.1, Mann–Whitney *U*-test). Three different filter sizes were used depending on patient’s body weight (small: 3–10 kg, medium: 10–30 kg, large: 30–60 kg). Most patients were in the post-operative period of a congenital cardiopathy. A summary of the patient characteristics is presented in Table 1, including the haemofilter settings. No patient experienced any adverse effect, including nephrotoxicity or hepatotoxicity, from the treatment.

**Table 1.** Summary of patient characteristics and haemofilter settings

	Without haemofilter (*n* = 15)^a^	With haemofilter (*n* = 12)^a^	*P* value^b^
Age (months)	7 (3–60)	20 (4–156)	0.11
Weight (kg)	8 (4.3–40)	9.11 (5.7–44)	0.26
Height (cm)	65 (53–131)	82.5 (60–146.5)	0.11
BSA (m^2^)	0.35 (0.24–1.18)	0.45 (0.29–1.32)	0.12
Haematocrit (%)	30.6 (20–38.7)	29.3 (26.3–37.5)	0.59
Haemoglobin (%)	9.7 (6.7–12.3)	9.8 (8.4–12.3)	0.71
Protein (g/dL)	5.1 (2.9–6.5)	5 (4–5.9)	1
Albumin (g/dL)	3.3 (2.1–4.2)	3.4 (2.5–4.2)	0.88
Creatinine (mg/dL)	0.32 (0.1–2.46)	—	—
eGFR (mL/min)	112 (29–283)	—	—
Male sex, %	75	40	0.07
Diagnosis cardiopathy, %	93.3	91.7	0.70
Post-operative period, %	60	83.3	0.24
Mechanical ventilation, *n* (%)	14 (93.3)	12 (100)	0.56
PRISM III score	6 (4–14)	8 (3.5–13.5)	0.95
Mortality, *n* (%)	3 (20)	5 (41.7)	0.39
Filter surface area (m^2^)			
* *Small (0.2)	—	*n* = 7	—
* *Medium (0.6)	—	*n* = 3	—
* *Large (1.2)	—	*n* = 2	—
Blood flow (mL/min)	—	50 (34–150)	—
Replacement fluid flow (mL/h)	—	95 (20–800)	—
Citrate flow^c^ (mL/h)	—	484 (273–1073)	
Dialysate flow (mL/h)	—	300 (100–1800)	—
Extraction flow (mL/h)	—	45 (30–120)	—
CKRT dose: total effluent flow (mL/kg/h)	—	56 (50–85)	—

Data are shown as the median (range), unless otherwise indicated.

^a^One patient without CKRT at the beginning of treatment required CKRT after the fifth dose, thus contributing to both groups.

^b^Mann–Whitney *U*-test.

^c^Pre-filter anticoagulation with citrate (18 mmol/L) was used in six patients.

### Brief description of the data

In total, 173 teicoplanin concentration measurements were used to develop the model. From these, 72 corresponded to patients without CKRT (plasma) and 101 to patients with CKRT (51 pre-filter and 50 post-filter samples). All concentration values were above the limits of quantification and ranged from 2.1 to 86.6 mg/L.

From the patients in the study, 16 of them (59%) reached the target trough concentration of 10 mg/L. Dividing by groups (with and without CKRT), the number of patients with and without CKRT reaching 10 mg/L was 5 (42%) and 11 (73%), respectively. For the AUC_24_/MIC > 800 target, assuming an MIC of 1 mg/L, the percentage of patients reaching the target *C*_min_ decreased to 0% and 3.7%, respectively, for each of the aforementioned groups.

### Population PK modelling

#### Base population model

The two-compartment model was selected (see Figure S2). The parameter CL_NR_ (non-renal clearance) could not be identified and was therefore removed from the model structure, an expected result since it is known from the literature that teicoplanin is eliminated exclusively by the kidneys.^16^ Data supported the estimation of IIV for the apparent volume of distribution of the central compartment (*V*_1_), renal clearance (CL_R_), and haemofilter clearance (CL_KRT_) (*P* < 0.01), but not for *V*_2_, the apparent volume of distribution of the peripheral compartment, and CL_D_, intercompartmental clearance (*P* > 0.05). Covariance between random effects was also non-significant (*P* > 0.05). Additional details are shown in the Supplementary material.

#### Covariate selection

Body weight impacted *V*_1_ and CL_R_ (*P* < 0.01) significantly and was incorporated in the model through the allometric expression using exponents of 1 and 0.75 for *V*_1_ and CL_R_, respectively. Conversely, including body weight as a covariate in *V*_2_ and CL_D_ did not improve model fit. Moreover, CL_KRT_ was significantly influenced by filter surface (*P* < 0.01). Effluent flow and blood flow were also tested as covariates affecting CL_KRT_. However, it was not possible to include them in the model along with filter size. No other patient characteristics or laboratory values, including estimated glomerular filtration rate (eGFR), significantly affected any of the PK parameters (*P* > 0.05), although CKRT parameters and haematocrit were included in the model (see more details in the Supplementary material).

**Figure 1.**
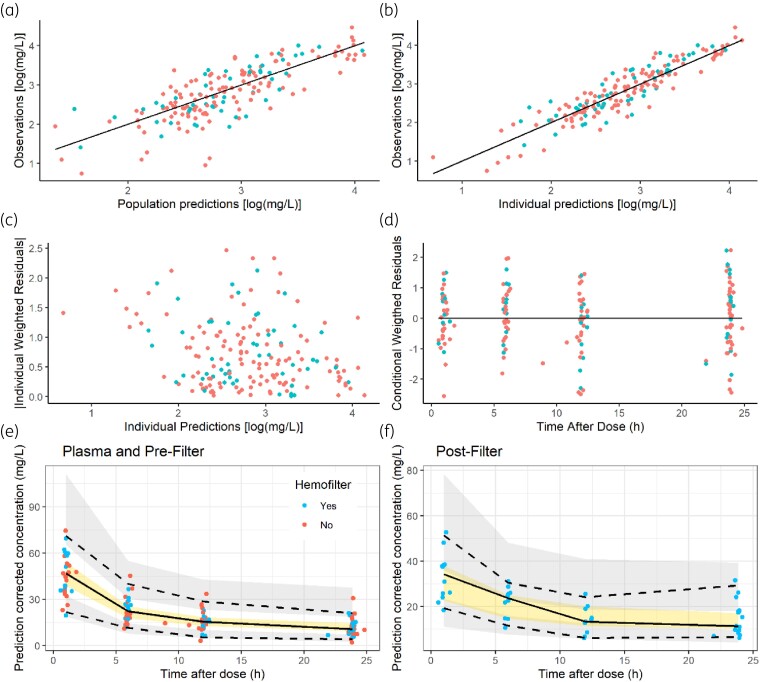
(a–d) Goodness-of-fit plots. Red points correspond to plasma and pre-filter samples, whereas blue points correspond to post-filter samples. Solid lines in black represent the perfect fit. (e–f) pcVPCs. Blue and red dots represent prediction-corrected observations from patients with and without CKRT, respectively. Dashed lines represent the 2.5% and 97.5% percentiles, whereas the solid lines represent the median of the prediction-corrected observations. Shadowed areas correspond to the 95% prediction intervals of 2.5%, 50% and 97.5% percentiles calculated from the population-predicted normalized simulated observations.

**Figure 2.**
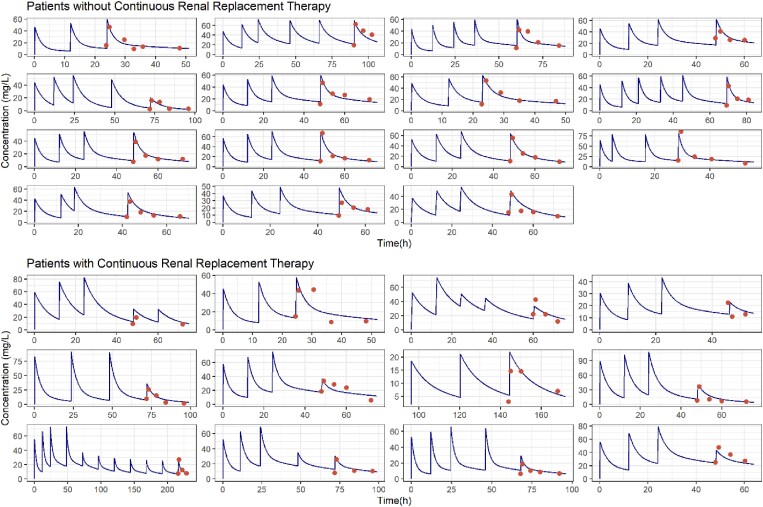
Individual-predicted plasma concentration profiles (blue lines) and observed plasma concentrations (red dots) versus time.

Table 2 lists the estimates of the parameters of the selected PK model. The corresponding values of relative standard error (RSE) (%) suggest that, in general, parameters were estimated with adequate precision. The absence of trends in the goodness-of-fit plots shown in Figure 1 reflect the lack of major model mis-specifications, and the low value of ε-shrinkage (Table 2) indicates that the observed-versus-individual plot is indeed informative with respect to model performance. Figure 2 represents the individual model-predicted profiles of *C*_Plasma_ and *C*_Pre_ during the complete treatment period together with the observed concentration values.

**Table 2.** Population PK parameter estimates corresponding to the selected model

Parameter	Parameter model	Estimates (RSE, %)	IIV (%) (RSE, %)	95% CI (SIR)	Shrinkage (%)
CL_R_ (L/h)^a^	$\theta_{CLR}\times {\left(\frac{WGT}{8}\right)}^{0.75}$	$\theta_{CLR}=0.169\;(12)$	30.4 (32)	0.139–0.208	33
CL_KRT_ (L/h)	$\theta_{CLKRT}\times \theta_{FILT}$	$\theta_{CLKRT}=0.119\;(8)$	14.1 (19)	0.103–0.136	45
		$\theta_{FILT\_Small}=1\;(Reference)$		—	
		$\theta_{FILT\_Med}=3.58\;(13)$		2.78–4.40	
		$\theta_{FILT\_Large}=5.04\;(14)$		3.80–6.26	
*V*_1_ (L)	$\theta_{V1}\times \left(\frac{WGT}{8}\right)$	$\theta_{V1}=1.56\;(17)$	39.2 (45)	1.03–1.98	40
*V*_2_ (L)	$\theta_{V2}$	$\theta_{V2}=3.03\;(30)$	—	2.10–4.53	—
CL_D_ (L/h)	$\theta_{CLD}$	$\theta_{CLD}=0.292\;(53)$	—	0.167–0.614	—
Residual error, log (mg/mL)	Plasma	0.301 (8)	—	0.27–0.34	9
	Post-filter	0.333 (13)		0.28–0.39	

WGT, weight. IIV is expressed as coefficient of variation (CV, %) calculated as $\sqrt{e^{\omega^{2}}-1}\times 100$, where $\omega^{2}$ corresponds to the variance of the random effects.

^a^CL_R_ in patients with CKRT was estimated to be zero.

#### Model evaluation

Results of the prediction-corrected visual predictive checks (pcVPCs) represented in Figure 1(e and f) show that the selected model captures properly the typical tendency and the dispersion of the three types of teicoplanin concentrations. The 95% CIs obtained from the sampling importance resampling (SIR) analysis and listed in Table 2 support parameter precision and suggest that the 95% CIs are not symmetric around the point estimates.

#### Probability of target attainment

**Figure 3.**
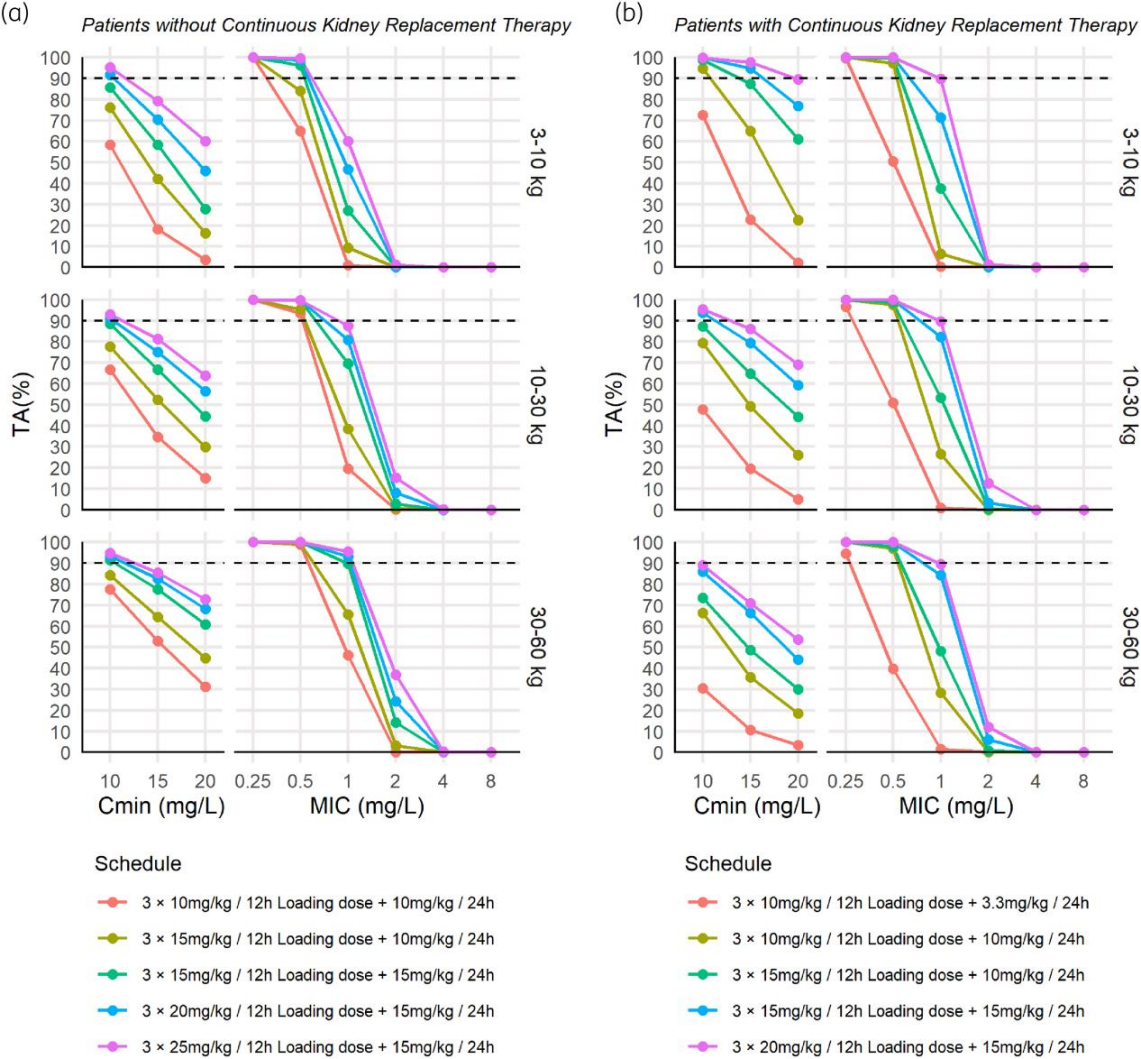
Probability of target attainment (%) for (a) *C*_min_ (>10, >15 or >20 mg/L) and (b) AUC_24_/MIC > 800 for each weight group. Different colours represent different dosing regimens. The current standard dosing regimen is represented in orange. Dashed lines correspond to a percentage of target attainment of 90%.

Figure S1 shows 95% prediction intervals of the simulated plasma concentrations corresponding to the different dosing scenarios explored highlighting the concentration thresholds for treatment efficacy and toxicity (10–60 mg/L, respectively). In simulations, the only covariate that was simulated was body weight, given that filter size was considered as a surrogate of body weight. Haemofilter settings were not needed to simulate plasma concentrations. Percentage of target attainment results are shown in Figure 3. In patients without haemofilter (Figure 3a), the current standard dosing regimen (red line) was clearly insufficient to achieve therapeutic concentrations. Similarly, in simulated patients undergoing CKRT (Figure 3b), the dosing regimen of 3 × 10 mg/kg/12 h + 3.3 mg/kg/24 h (red line) was also insufficient to reach therapeutic concentrations in many cases.

## Discussion

Although teicoplanin has been widely used in critically ill patients, few population PK studies have been performed in the paediatric population,^2,9,17,18^ and none in paediatric patients undergoing CKRT. In this study, pharmacokinetics of teicoplanin have been characterized in paediatric patients with and without CKRT using a population PK model. A two-compartment model was selected, resulting in an acceptable precision of parameter estimates.

In general, the typical estimates of the model parameters are in accordance with those already published in patients not requiring CKRT. For example, the estimate of the renal clearance of 0.169 L/h fell within the 0.105–0.176 L/h range published elsewhere.^2,9,17,18^ The estimates of *V*_1_ (1.56 L) and *V*_2_ (3.03 L) are also similar to the corresponding values reported in the literature, (2.07–2.28 L, and 3.70 and 15.5 L, respectively).^2,17,18^ In terms of clearance during kidney replacement therapy, the estimate of CL_KRT_ (0.119 L/h) was 30% lower than CL_R_, suggesting that patients with a haemofilter might need lower doses.

The only patient characteristic that showed significant covariate effects was body weight on CL_R_ and *V*_1_ in consonance with published results.^2,17,18^ Note that renal function expressed as eGFR was not found to have an impact on CL_KRT_, consistent with the results of Aulin *et al*.^19^ This may be explained by the relatively reduced sample size and number of observations in each GFR category, especially those with reduced renal clearance. The model predicts an increase in 0.016 L/h and 0.19 L, respectively, for an increment of 1 kg of body weight. Our analysis did not find significant differences in the apparent volumes of distribution between patients with or without CKRT, in agreement with previous results with other antibiotics. Similarly to other recent reports, the size of the haemofilter significantly impacted the magnitude of the extracorporeal elimination.^6^ Effluent flow and blood flow did not improve model fit due to strong correlations with filter size (Figure S3). Therefore, it was not possible to include these covariates simultaneously in CL_KRT_. The incorporation of body weight in the model decreased the unexplained IIV in CL_R_ and *V*_1_ from 77% to 30% and 160% to 39%, respectively. For the case of CL_KRT_, the size of the filter accounted for the 82% of unexplained variability. The population (0.119 L/h) and individual estimates of CL_KRT_ (0.099–0.598 L/h) agree well with the high protein binding where theoretically only 10% of the drug should be eliminated. Interestingly, albumin concentration did not show covariate effects in any of the model parameters despite the fact that teicoplanin is highly bound to that plasma protein. The same results were obtained previously in the work of Aulin *et al*.,^19^ where the patient population showed a range of albumin concentrations very similar to that of our subjects. It is possible that the reduced sample population and the narrow range of albumin distribution jeopardize the impact of albumin plasma levels on the pharmacokinetics of teicoplanin. Observed and predicted serum concentrations are, in most cases, lower than the estimate of 64.2 mg/L reported by Aulin *et al.*, for the parameter K_D_, the dissociation constant of the drug–albumin complex, indicating linear binding kinetics.

Starting teicoplanin therapy with appropriate loading doses is mandatory for rapidly achieving therapeutically effective concentrations from the beginning of treatment.^3^ The conducted simulations with the standard dosing regimen show that three loading doses of 10 mg/kg every 12 h followed by 10 mg/kg every 24 h (3.3 mg/kg in CKRT patients) are insufficient to achieve therapeutic concentrations in patients both with and without CKRT, due to the low calculated percentage of target attainment for the traditional target of *C*_min _> 10 mg/L (a mean of 84% for simulated patients with a haemofilter and 66% without a haemofilter). Other studies have also found the need for increasing the dosage in paediatric patients.^2,8,9^ Therefore, different alternatives involving higher doses are warranted.

Additionally, teicoplanin is considered an antibiotic with a low level of side effects, which is why it is preferred to vancomycin when immunocompromised or paediatric patients have to be treated. It is generally accepted that a trough concentration of 60 mg/L should not be exceeded.^15^ However, recent studies indicate that a lower threshold may help reduce toxicity,^20^ although further studies are needed to confirm this. In our case, we aimed to achieve a median trough concentration above 15 and up to 20 mg/L for treating severe infections.

In critically ill patients with normal renal function, simulation results suggest that to achieve a percentage of target attainment of >90% above a *C*_min_ of 10 mg/L, an increase in loading doses to 15 mg/kg may be beneficial (Figure 3a, green lines). Additionally, in patients weighing less than 30 kg with severe infections, to achieve a percentage of target attainment of >90% above a *C*_min_ of 15 mg/L, consideration should be given to increasing the maintenance dose to 15 mg/kg (Figure S1a, central column). In patients undergoing haemofiltration, the daily dose should be maintained at least at 10 mg/kg, to achieve a percentage of target attainment of >90% above a *C*_min_ of 10 mg/L, particularly in patients weighing less than 10 kg. Additionally, as observed in Figure S1, to prevent *C*_min _> 60 mg/L, a loading dose exceeding 15 mg/kg in patients weighing more than 10 kg undergoing haemofiltration should be avoided, especially in the absence of real-time drug measurements.

Our results indicate that the clearance of teicoplanin elicited by the haemofiltration device is efficient despite its high protein-binding affinity. A significant factor in paediatric patients may be the high total effluent dose required, particularly when citrate is used as an anticoagulant. In the PK study conducted in adults by Chen *et al*.,^1^ the recommended dosage suggests that when the total effluent flow rate of CKRT exceeds 35 mL/kg/h, as in the present analysis, the maintenance dose should be increased by 30%, bringing it to at least 8 mg/kg/day.^10^ However, we did not explore different dosing intervals to maintain clinical feasibility. This decision was made considering one of the main advantages of teicoplanin, its relatively long half-life, allowing once-daily dosing.^3^

Despite the data analysed supporting the model structure and the precise estimation of corresponding parameters, the dosing recommendations provided in this evaluation should be considered with caution, given the small sample size and the fact that most subjects were post-operative congenital heart disease patients. In order to generalize our results a larger and more heterogeneous patient population is required. Nevertheless, our work can be used in the design of dedicated PK studies. In addition, the use of therapeutic drug monitoring has been strongly advocated for the use of teicoplanin in critically ill patients,^2,11,19^ and we postulate that the patients with CKRT will benefit as well from that approach, although dedicated studies are required.

## Limitations

Limitations exist within this study, primarily due to the small sample size, inherent to all paediatric studies involving this type of patient populations, which might have hampered the identification of covariate effects such as eGFR on CL_R_ (not relevant in the case of children with continuous renal replacement therapy). Moreover, eGFR values were calculated using the bedside Schwarz equation due to missing Cystatin C measurements in 27% of the patients. Neonatal patients, less than 28 days of postnatal age, were not included in this study. One additional limitation of the present study is the absence of measurements in the effluent which could have helped to evaluate possible adsorption of teicoplanin in the haemofilter. Lastly, variations in haemofilter settings across centres may cause discrepancies. Additionally, although real-time therapeutic drug monitoring (TDM) is crucial, the delay in retrieving results can sometimes compromise the safety and accuracy of this type of PK model.

### Conclusions

To summarize, our PK model can help in selecting adequate teicoplanin doses in paediatric patients, highlighting body weight and filter size as significant factors. Current standard dosing may be insufficient; we propose 3 × 15 mg/kg/12 h, then 15 mg/kg daily for non-CKRT children, and 3 × 15 mg/kg/12 h, then 10 mg/kg daily for CKRT children to achieve > 90% percentage of target attainment (*C*_min_ > 15 mg/L). Individualization of teicoplanin treatment based on real-time therapeutic drug monitoring and dosing adaptation may represent an important tool in antimicrobial stewardship programmes.

## Supplementary Material

dkaf012_Supplementary_Data
