# Supplementary material for: Population pharmacokinetic analysis of teicoplanin in paediatric patients, including those receiving continuous kidney replacement therapy: a prospective cohort study
Source: J Antimicrob Chemother. 2025 Jan 23;80(3):868–75. doi: 10.1093/jac/dkaf012 (PMC11879232; doi:10.1093/jac/dkaf012)
Supplement: dkaf012_Supplementary_Data [file dkaf012_supplementary_data.docx]

**Supplementary material**

1. **Patient infections**

Patients were empirically treated for suspected catheter-related bacteremia in 40.7 % of cases, with confirmation of Gram-positive bacteria in 3 patients. In 25,9 % of cases, empiric therapy was administered for ventilator-associated pneumonia and 18,5 % mediastinitis. In 4 cases, treatment was initiated due to sepsis in patients with extracorporeal devices and/or risk of endocarditis.

1. **Analytical method**

Samples were analyzed at Reference laboratory in Barcelona, Spain, using a validated liquid chromatography/tandem mass (LS-MS/MS) method to quantify teicoplanin concentrations. UPLC composed of 1290 Bin Pump and 1290 Multisampler from Agilent Technologies coupled to Agilent Technologies 6430 QQQ Tandem Mass detector. The chromatographic column was Zorbax Eclipse plus C18, 1.7 microns. Teicoplanin standard and quality controls were used for calibration and ensuring precision. Protein precipitation in plasma and urine samples was conducted with acetonitrile (1:1) and subsequent dilution of the supernatant with milli-Q water (1:1). Subsequent filtering using a 0.2 micron filter. Mobile phases (chromatographic gradient): Water (0.1% Formic Acid) – Methanol (0.1% Formic Acid). Total chromatogram time: 6 minutes. Injection volume: 8 microliter. Linearity was established over the concentration range from 0 to 100 mcg/mL. The limit of quantification (LOQ) was set at 1.0 mg/l. Calibration standard: Teicoplanin T0578 from Sigma. Coefficient of variation (precision): 7%.

1. **Pharmacokinetic analysis**

The selection of the optimal candidate model was based on various criteria. Firstly, a significant reduction of 3.84 points in the objective function (-2×LogLikelihood) between two hierarchical models, with significance determined at 5% for differences in a single parameter. Secondly, precision of the parameters was assessed using the relative standard error expressed as a percentage, which was calculated as the standard error divided by the estimate of the corresponding parameter, multiplied by 100. And lastly, the visual exploration of the goodness of fit plots.

*Model building*. Model building started with the development of the base population model, proceeded with covariate selection, and concluded with model evaluation.

*Base population model*. Plasmatic, prefilter and postfilter concentrations were modelled simultaneously using compartmental models, which were parameterized in terms of apparent volumes of distribution (V1 and V2), and inter-compartmental (CLD) and elimination (CL) clearances, the latter comprising the contribution of different potential elimination mechanisms including the renal (CLR), non-renal (CLNR) and extracorporeal (CLKRT).

In this work, we have dismissed renal clearance (CLR) in patients with CKRT, as our analyzed data suggested that there was no renal elimination in these patients, thus, CL equals CLKRT. Postfilter concentrations were modelled as a function of CPre and CLKRT according to the expression provided in supplementary material.

Corrections for measured prefilter and postfilter concentrations were made in the model taking into account the different flow supplies and removals (see Table 1), as suggested by Broeker et al., 2020 [1]. To take into account the different inflows and outflows around the CKRT, the following corrections were applied in the model:

$$C_{Pre Corr}=C_{Pre Meas}\times\frac{\varphi_{Pl Corr}+\varphi_{Citrate}}{\varphi_{Pl Corr}}$$

$$C_{Post Corr}=C_{Post Meas}\times\frac{\varphi_{Pl Corr}-\varphi_{Filt}}{\varphi_{Pl Corr}}$$

Where C_Pre Corr_ and C_Post Corr_ mean the corrected prefilter and postfilter concentrations, C_Pre Meas_ and C_Post Meas_ mean the measured prefilter and postfilter concentrations. ϕ_Pl Corr_ refers to the corrected plasma flow that goes into the hemofilter, which is calculated as ϕ_Blood_ × BPR (blood to plasma ratio). ϕ_Citrate_ corresponds to the citrate supply flow and ϕ_Filt_ corresponds to the plasma flow that is lost in the filter and goes into the effluent. All flows are expressed in L/h.

Postfilter concentrations were modelled as a function of C_Pre_ and CL_KRT_ in the model with the following expression:

$$C_{post}=C_{pre}\times\left( 1-\frac{{CL}_{KRT}}{\varphi_{Pl Corr}} \right)$$

For each individual, all the flow values (ϕ) remained constant during the study.

*Covariate selection*. More than 10 covariates (Table 1) including age, weight, height, eGFR (calculated with Schwartz formula), filter surface, albumin, creatinine, hematocrit, hemoglobin, urea, and body surface area were studied and their relationships with model parameters were evaluated. Linear and nonlinear correlations were explored in all parameters according to biological plausibility and covariates were considered significant using 0.05 and 0.01 levels of significance during the forward inclusion and backward deletion procedures, respectively.

*Model evaluation*. The prediction corrected visual predictive checks (pcVPCs) [2] were conducted. One thousand studies of the same design characteristics of the original one were simulated using the selected model and its corresponding parameters. For each simulated dataset and time bin the 5th, 50th, and 95th percentiles of the simulated concentrations normalized the by the corresponding population prediction were calculated. Then, the 95% prediction intervals of the aforementioned percentiles were obtained and represented graphically together with the percentiles of the normalized observed concentrations. Parameter precision was further evaluated calculating the 95% confidence intervals with the sampling importance re-sampling method [3].

1. **Software**

SIR results were obtained with PsN [4, 5], Pirana package [6] and RStudio 2023.12.0 [7] were used to process raw data and NONMEM results and generate the required graphics for model selection and evaluation.

1. **Results**

The one-compartment model performed significantly worse than the two-compartment model (p<0.01). On the other hand, the three-compartment model did not improve the fit significantly with respect the two-compartment model (p>0.05). The estimates of the fixed effect parameters were 1.49 L, 2.97 L, 0.29 L/h, 0.17 L/h and 0.11 L/h for V_1_, V_2_, CL_D_, CL_R_, and CL_KRT_, respectively. With respect random effects the estimates of IIV expressed as coefficient of variation [CV(%)] were 42.5, 29.3, and 12.9 for V_1_, CL_R_, and CL_KRT_, respectively.

**Table S1**. Dosing regimens used for simulations

|  | **Simulated dosing regimens** |
| --- | --- |
| **Patients without CKRT** | 3 × 10mg/kg / 12h Loading dose + 10mg/kg / 24h |
|  | 3 × 15mg/kg / 12h Loading dose + 10mg/kg / 24h |
|  | 3 × 15mg/kg / 12h Loading dose + 15mg/kg / 24h |
|  | 3 × 20mg/kg / 12h Loading dose + 15mg/kg / 24h |
|  | 3 × 25mg/kg / 12h Loading dose + 15mg/kg / 24h |
| **Patients with CKRT** | 3 × 10mg/kg / 12h Loading dose + 3.3mg/kg / 24h |
|  | 3 × 10mg/kg / 12h Loading dose + 10mg/kg / 24h |
|  | 3 × 15mg/kg / 12h Loading dose + 10mg/kg / 24h |
|  | 3 × 15mg/kg / 12h Loading dose + 15mg/kg / 24h |
|  | 3 × 20mg/kg / 12h Loading dose + 15mg/kg / 24h |

Doses were administered via intravenous infusion during 5 minutes.


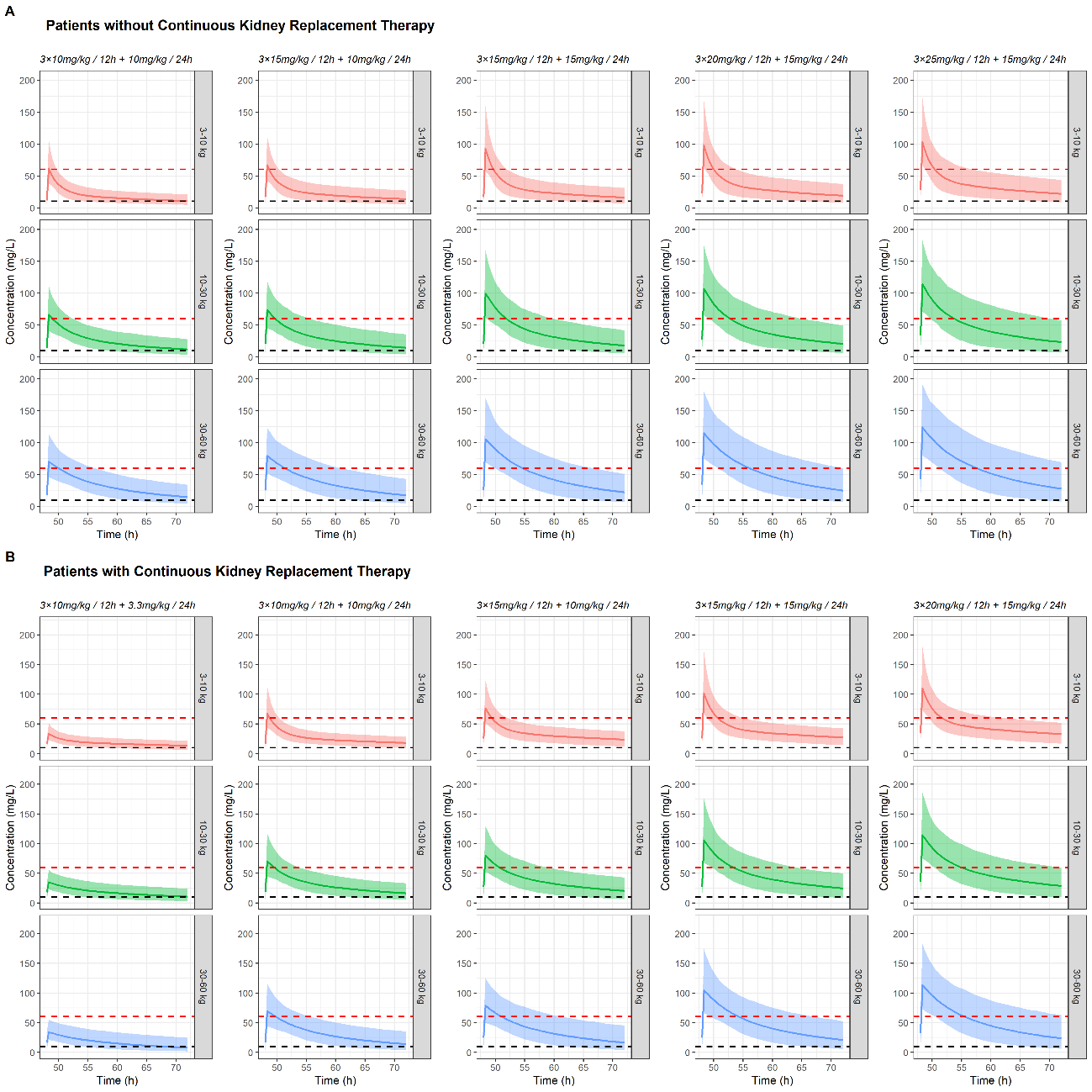


**Figure S1.** Simulated plasma and prefilter concentrations for the 48-72h period after the first dose. Solid lines represent the median of the concentrations. Shadowed areas represent the 95% prediction interval of the concentrations. Dashed lines in black and red cover the therapeutic range (10-60 mg/L).


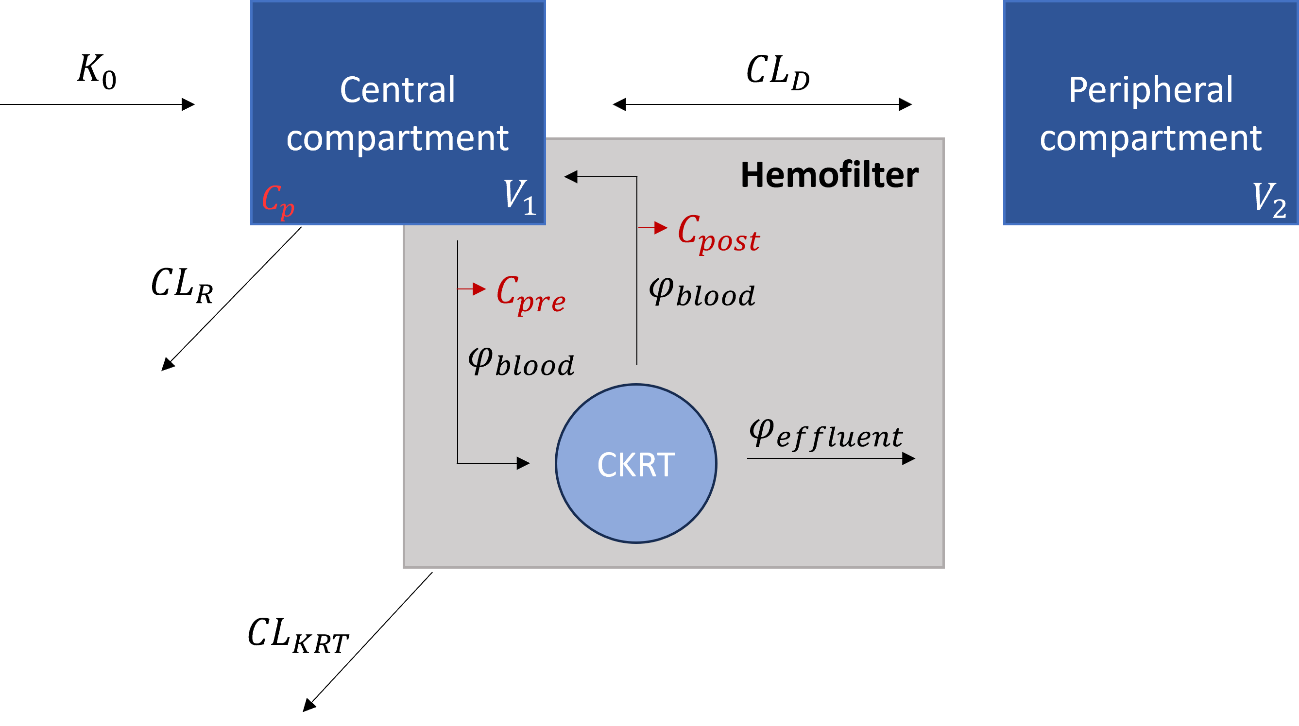


**Figure S2.** Model structure. K_0_, infusion rate; CL_D_, distribution clearance; CL_R_, renal clearance; CL_KRT_, hemofilter clearance; CKRT, Continuous kidney replacement therapy; ϕ_blood_, blood flow; ϕ_effluent_, effluent flow; C_p_, teicoplanin plasma concentration; C_pre_, teicoplanin prefilter concentration; C_post_, teicoplanin postfilter concentration.


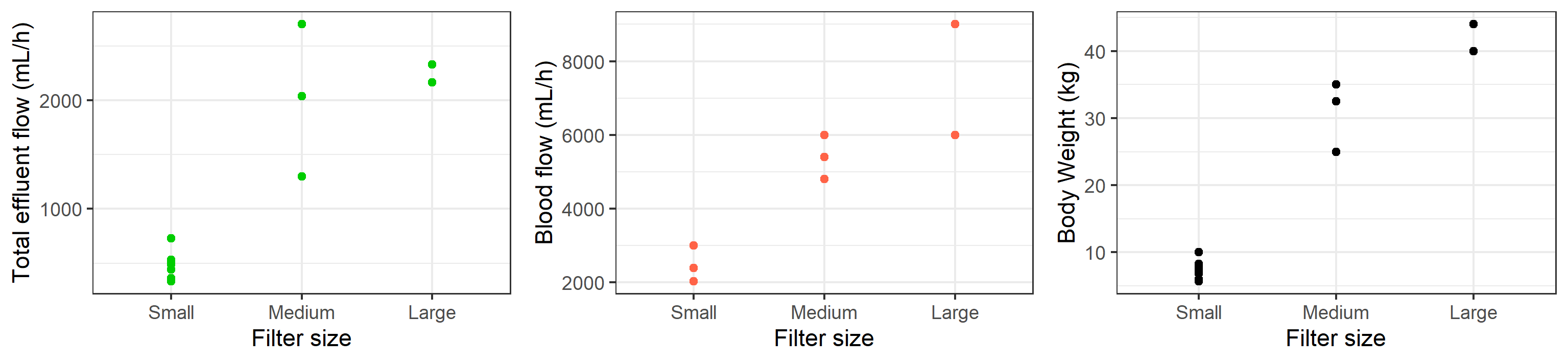


**Figure S3**. Individual hemofilter settings are represented with points for patients receiving CKRT.

**Supplementary material references**

[1] Broeker, A., Vossen, M. G., Thalhammer, F., Wallis, S. C., Lipman, J., Roberts, J. A., & Wicha, S. G. (2020). An Integrated Dialysis Pharmacometric (IDP) Model to Evaluate the Pharmacokinetics in Patients Undergoing Renal Replacement Therapy. Pharmaceutical research, 37(6), 96. <https://doi.org/10.1007/s11095-020-02832-w>

[2] Nguyen, T. H., Mouksassi, M. S., Holford, N., Al-Huniti, N., Freedman, I., Hooker, A. C., John, J., Karlsson, M. O., Mould, D. R., Pérez Ruixo, J. J., Plan, E. L., Savic, R., van Hasselt, J. G., Weber, B., Zhou, C., Comets, E., Mentré, F., & Model Evaluation Group of the International Society of Pharmacometrics (ISoP) Best Practice Committee (2017). Model Evaluation of Continuous Data Pharmacometric Models: Metrics and Graphics. CPT: pharmacometrics & systems pharmacology, 6(2), 87–109.

[3] Dosne, A. G., Bergstrand, M., Harling, K., & Karlsson, M. O. (2016). Improving the estimation of parameter uncertainty distributions in nonlinear mixed effects models using sampling importance resampling. Journal of pharmacokinetics and pharmacodynamics, 43(6), 583–596.

[4] Lindbom L, Ribbing J, Jonsson EN. Perl-speaks-NONMEM (PsN) - A Perl module for NONMEM related programming. Comput Methods Programs Biomed. 2004 Aug 1;75(2):85–94.

[5] Lindbom L, Pihlgren P, Jonsson N. PsN-Toolkit - A collection of
computer intensive statistical methods for non-linear mixed effect modeling
using NONMEM. Comput Methods Programs Biomed. 2005 Sep 1;79(3):241–57.

[6] R Core Team (2023). _R: A Language and Environment for Statistical Computing_. R Foundation for Statistical Computing, Vienna, Austria. <https://www.R-project.org/>.

[7] Posit team (2023). RStudio: Integrated Development Environment for R. Posit Software, PBC, Boston, MA. URL <http://www.posit.co/>.
